# Dietary Niacin Requirement of Juvenile Chinese Mitten Crab, *Eriocheir sinensis*

**DOI:** 10.1155/2022/8348000

**Published:** 2022-10-28

**Authors:** Xuran Liu, Wenlong Wan, Mengge Li, Jiayuan Shi, Jie Xu, Zihan Zhou, Anran Wang, Shuyan Miao

**Affiliations:** College of Animal Science and Technology, Yangzhou University, 48 Wenhui East Road, Yangzhou 225009, China

## Abstract

Effects of dietary niacin on the growth performance, intestinal histomorphology, body composition, and antioxidant capacity were investigated in the present study to determine the optimum requirement of niacin for juvenile *Eriocheir sinensis*. All 360 crabs (initial average weight 1.14 ± 0.04 g) were randomly divided into 6 groups with 3 replicates in each group and 20 crabs in each replicate. Crabs were fed with the control diet (0.89 mg/kg) or niacin-supplemented diets (170.54 mg/kg, 347.05 mg/kg, 587.59 mg/kg, 784.85 mg/kg, and 1248.86 mg/kg) for 12 weeks (named as G1, G2, G3, G4, G5, and G6, respectively). The results showed that appropriate dietary niacin (above 347.05 mg/kg) significantly increased the weight gain rate (WGR) and specific growth rate (SGR) (*p* < 0.05), but did not affect the survival rate (SR), feed conversion ratio (FCR), daily feeding rate (DFR), and molting frequency (MF) of crabs (*p* > 0.05). The niacin content in the hepatopancreas of crabs in G1 and G2 was significantly lower than that of the other four groups (*p* < 0.05). Moreover, dietary niacin significantly affected the intestinal histomorphology of crabs, including the number of folds (NF), height of folds (HF), height of microvillus (HMV), and thickness of muscularis (TM) (*p* < 0.05). Additionally, moderate dietary niacin levels significantly affected the nonspecific immune responses of crabs, by improving the activity of catalase (CAT), glutathione s-transferase (GST), glutathione peroxidase (GSH-Px), and total superoxide dismutase (T-SOD) (*p* < 0.05). Based on the broken-line model analysis of SGR against dietary niacin level, the dietary niacin requirement of juvenile crabs was suggested to be 419.4 mg/kg.

## 1. Introduction

Niacin is a water-soluble vitamin with stable physicochemical properties. It can act as a precursor of cellular metabolic coenzymes, including nicotinamide adenine dinucleotide (NAD^+^) and nicotinamide adenine dinucleotide phosphate (NADP^+^), which are involved in lipid metabolism, respiratory oxidation, anaerobic catabolism of sugars, and so on [1]. Inadequate intake of niacin may lead to several deficiency symptoms in crustaceans, such as anorexia and aversion to feed. Prolonged niacin deficiency caused death with the blackening of gills in some shrimp [2]. Meanwhile, excessive niacin intake has been reported to negatively affect the feed utilization and growth performance of juvenile grass shrimp (*Penaeus monodon*) and Pacific white shrimp (*Litopenaeus vannamei*) [3, 4]. Therefore, it is important to investigate the appropriate dietary supplementation of niacin in aquatic animals for optimum growth and feed utilization.

Studies on niacin utilization have been conducted in several fish species, including Atlantic salmon (*Salmo salar*) (150-200 mg/kg) [5], juvenile Jian carp (*Cyprinus carpio* var. Jian) (24.90 mg/kg) [6], juvenile grass carp (*Ctenopharyngodon idella*) (25.5 mg/kg) [7], adult GIFT tilapia (*Oreochromis niloticus*) (63.62 mg/kg) [8], juvenile Kunming schizothoracin (*Schizothorax grahami*) (36.83 mg/kg) [9], and juvenile golden pompano (*Trachinotus ovatus*) (29.85-32.25 mg/kg) [10], with a wide variation in dietary requirement for different species of fish. Also, the following results of shrimps have been found in kuruma prawn (*Penaeus japonicus*) (400 mg/kg) [11], juvenile grass shrimp (7.18 mg/kg) [3], Chinese prawn (*Penaeus chinensis*) (400 mg/kg) [12], Indian white prawn (*Penaeus indicus*) (250 mg/kg) [2], and juvenile Pacific white shrimp (109.55 mg/kg) [4]. Up to now, the niacin requirements of crabs have been rarely reported.

Chinese mitten crab, *Eriocheir sinensis*, is a valuable economic crab species and popularly cultured in China. The vitamin nutritional requirements of C, B_6_, B_2_, B_12_, B_9_, E, D_3_, and A [13–20] have been reported in *E. sinensis*. The present study was conducted to investigate the effects of dietary niacin on growth performance, body composition, intestinal histomorphology, and antioxidant capacity of crabs and then to determine the optimum niacin requirement of *E. sinensis*.

## 2. Materials and Methods

### 2.1. Ethical Approval

All experiments and animal care were approved by the Yangzhou University Institutional Animal Use and Care Committee.

### 2.2. Experimental Diets

The formulation and basal nutrient compositions are shown in Table 1. Casein (vitamin-free) was used as the main protein source. Fish oil, soybean oil, and soy lecithin were used as the main lipid sources. Different levels of niacin (99%, Shanghai Aladdin Biochemical Technology Co., Ltd., China; 0 mg/kg, 200 mg/kg, 400 mg/kg, 600 mg/kg, 800 mg/kg, and 1200 mg/kg) were added to produce six experimental diets, which were named as D1, D2, D3, D4, D5, and D6, respectively.

Diets were prepared according to the method used by Miao et al. [21]. All dry ingredients were ground through a 100-mesh screen and thoroughly mixed with a Hobart-type mixer (VH10, Shanghai Tian He Machinery Equipment Co., Ltd), and then, fish oil, soybean oil, and distilled water were added to produce cold-extruded pellets (1.0 mm and 1.5 mm in diameter, 3 mm in length) with a twin-screw extruder (F-26 III, South China University of Technology, Guangzhou). All diets were air-dried (60°C) and stored (-20°C). The actual contents of dietary niacin were 0.89 mg/kg, 170.54 mg/kg, 347.05 mg/kg, 587.59 mg/kg, 784.85 mg/kg, and 1248.86 mg/kg, respectively; the niacin content in diets was determined according to Mawatari et al. [22].

### 2.3. Experimental Animals and Conditions

*E. sinensis* was purchased from a commercial farm (Yangzhou, China). Prior to the initiation of the feeding trial, all crabs were acclimated in two cement ponds with a recirculating system in the greenhouse of Yangzhou University and fed with commercial feed (35% crude protein and 6% lipid) at 08:00 and 17:00 every day. After 2-week acclimation, 360 healthy crabs (initial weight 1.14 ± 0.04 g) were subjected to fasting for 24 h and then randomly assigned to 6 groups, with 3 replicated square plastic buckets in each group and 20 crabs in each bucket (75 × 56 × 40 cm, *L* : *W* : *H*). Four arched tiles were placed in each bucket to prevent them from fighting each other. Crabs were fed with experimental diets 3 times daily, 08:00 (40%), 12:00 (20%), and 16:00 (40%) at a ratio of 4% of their average body weight for 12 weeks. One hour after feeding, the residual diet was collected, dried, and weighed. 30% of the water in each bucket was exchanged daily. The water quality was determined once a week. The nitrite-N and total ammonia nitrogen were at negligible levels, water temperature ranged from 24.0 to 27.0°C, and the dissolved oxygen concentration was 6.7-7.2 mg/L.

### 2.4. Sample Collection and Analysis

In the culture experiment, the number of shells shed by crabs in each bucket was collected and recorded for the calculation of molting frequency (MF). After the feeding trial, all crabs were subjected to fasting for 24 h, anesthetized on ice, weighed, and counted to calculate growth performance, feed utilization, and survival rate. Then, 10 crabs were randomly sampled from each bucket; the muscle, hepatopancreas, and intestines samples were collected and stored at -80°C.

Proximate compositions of the ingredients and diets were analyzed according to the standard AOAC method [23]. Briefly, moisture was analyzed by oven drying to a constant weight at 105°C, crude protein was measured using the Kjeldahl method, crude lipid was analyzed by the Soxhlet extraction method, and crude ash was determined by combustion in a muffle furnace at 550°C for 4 h.

After 24 h of fixation, the intestines of three crabs in each bucket were sampled; the digesta was squeezed out; and then, the midintestines were stored in Bouin's fixative. The distal intestinal morphology was observed by H&E staining of the midintestine [24]. Four morphological parameters including the number of folds (NF), height of fold (HF), thickness of muscularis (TM), and height of microvillus (HMV) were determined using the method implemented by Hu et al. [25].

The content of niacin in the hepatopancreas was determined by the method of Mawatari et al. [22]. The immune-related enzymes and antioxidant indexes in the hepatopancreas, including the activity of acid phosphatase (ACP), alkaline phosphatase (AKP), total superoxide dismutase (T-SOD), glutathione peroxidase (GSH-Px), glutathione s-transferase (GST), catalase (CAT), total antioxidant capacity (T-AOC), and malondialdehyde (MDA) value, were determined using commercial kits (Jiancheng Bioengineering Institute, Nanjing, China). The specific operation and calculations were conducted according to the manufacturer's instructions.

### 2.5. Calculations and Statistical Analysis

The weight gain rate (WGR), specific growth rate (SGR), feed conversion ratio (FCR), survival rate (SR), molting frequency (MF), and daily feeding rate (DFR) were calculated according to the following formula:

(1) $$\begin{array}{c} \text{Weight gain rate }\left(\text{WGR},\%\right)=100\times \frac{\text{final body weight}-\text{initial body weight }}{\text{initial body weight}},\\ \text{Specific growth rate }\left(\text{SGR},\frac{\%}{\text{d}}\right)=100\times \frac{\ln\;\left(\text{final body weight}\right)-\ln\;\left(\text{initial body weight}\right)}{\text{breeding days}},\\ \text{Survival rate }\left(\text{SR},\%\right)=100\times \frac{\text{number of surviving crabs}}{\text{initial number of crabs}},\\ \text{Feed conversion ratio }\left(\text{FCR}\right)=\frac{\text{weight of feed consumed by the crabs}}{\text{final body weight}-\text{initial body weight}},\\ \text{Molting frequency }\left(\text{MF},\%\right)=100\times \frac{\text{total number of the molting times per bucket}}{\text{initial number of crabs}},\\ \text{Daily feeding rate }\left(\text{DFR},\%\right)=100\times \frac{\text{total dry feed intake}}{\text{average wet weight at initial and final}\times \text{days fed }}. \end{array}$$

### 2.6. Statistical Analysis

All data were statistically analyzed using Excel 2010 and SPSS 25.0 (SPSS Inc., Chicago, USA) software. Homogeneity of variance was checked before a one-way analysis of variance (ANOVA), followed by Tukey's multiple comparison test to assess the significant differences among the groups with data expressed as mean ± standard deviation (means ± S.D.) (significance level *p* < 0.05). In addition, to determine if the effect was linear and/or quadratic, a follow-up trend analysis using orthogonal polynomial contrasts was performed [26]. The optimum requirement of juvenile *E. sinensis* for niacin was estimated by broken-line regression analysis [27].

## 3. Results

### 3.1. Growth Performance and Feed Utilization

**Figure 1:**
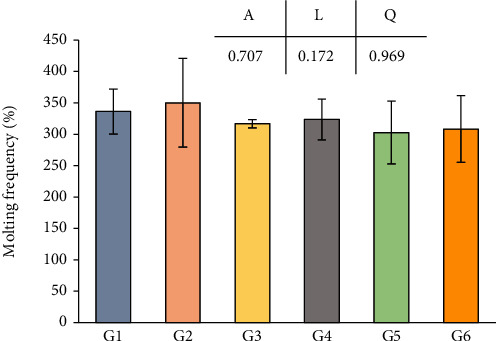
Effects of dietary niacin levels on molting frequency of *Eriocheir sinensis*. The values are the means ± S.D. (*n* = 3). Different letters indicate significant differences (*p* < 0.05). *A*: ANOVA; *L*: linear trend; *Q*: quadratic trend.

**Figure 2:**
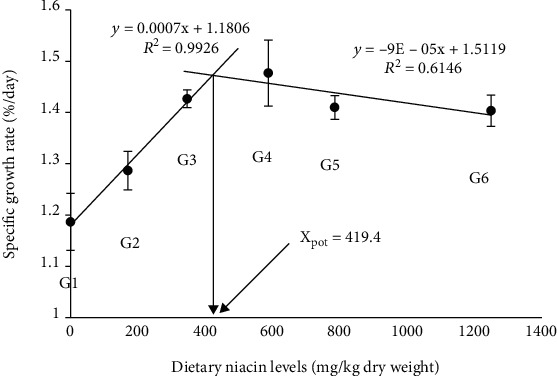
Relationship between specific growth rate and dietary niacin levels based on a two-slope broken-line regression analysis, where *X*_pot_ represents the optimal dietary niacin level for the maximum growth of *Eriocheir sinensis*. The values are the means ± S.D. (*n* = 3).

Effects of dietary niacin levels on the growth performance, SR, and feed utilization of crabs are shown in Table 2. No significant difference was found in SR, FCR, and DFR among all treatments (*p* > 0.05). However, dietary niacin supplementation significantly affected the final weight, WGR, and SGR of crabs (*p* < 0.05). With the dietary niacin increased from 0.89 to 347.05 mg/kg, the final weight and SGR significantly increased (*p* < 0.05), while no significant difference in WGR was found in crabs between G1 and G2 (*p* > 0.05). Meanwhile, when the dietary niacin ranged from 347.05 to 1248.86 mg/kg, no significant difference in final weight, WGR, and SGR was found among G3, G4, G5, and G6 (*p* > 0.05). There were significantly positive linear and negative quadratic trends between the dietary niacin levels and the dependent variables including the final weight, WGR, and SGR. Additionally, the MF also showed no significant difference among all groups (Figure 1). To further determine the optimal supplementation dosage of niacin, a broken-line regression analysis of SGR against dietary niacin levels was conducted, which indicated that the optimal dietary niacin level of juvenile *E. sinensis* was estimated to be 419.4 mg/kg diet (Figure 2).

### 3.2. Niacin Deposition

**Figure 3:**
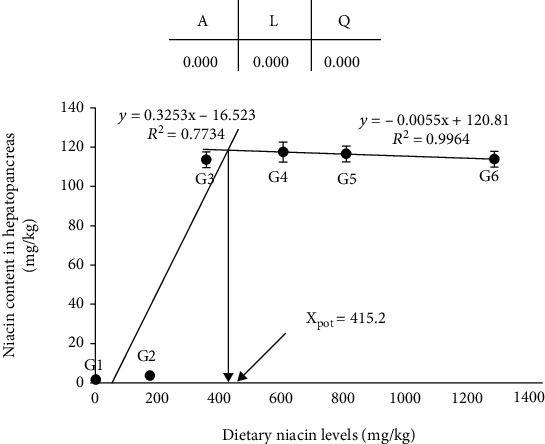
Relationship between niacin content in the hepatopancreas and dietary niacin levels based on a two-slope broken-line regression analysis, where *X*_pot_ represents the optimal dietary niacin level for the maximum deposition of niacin in the hepatopancreas of *Eriocheir sinensis*. The values are the means ± S.D. (*n* = 3). *A*: ANOVA; *L*: linear trend; *Q*: quadratic trend.

Effects of dietary niacin levels on the niacin content in the hepatopancreas are shown in Figure 3. There was no significant difference in niacin content in the hepatopancreas of crabs between G1 and G2, as well as between G3, G4, G5, and G6 (*p* > 0.05). However, the niacin content in G1 and G2 was significantly lower than that in the other four groups (*p* < 0.05). Linear trend and quadratic trend were found between the dietary niacin levels and niacin content in the hepatopancreas. Based on the hepatopancreas niacin content against the dietary niacin level analyzed by broken-line regression, the optimal dietary niacin level of juvenile *E. sinensis* was suggested to be 415.2 mg/kg diet.

### 3.3. Intestinal Histomorphology

**Figure 4:**
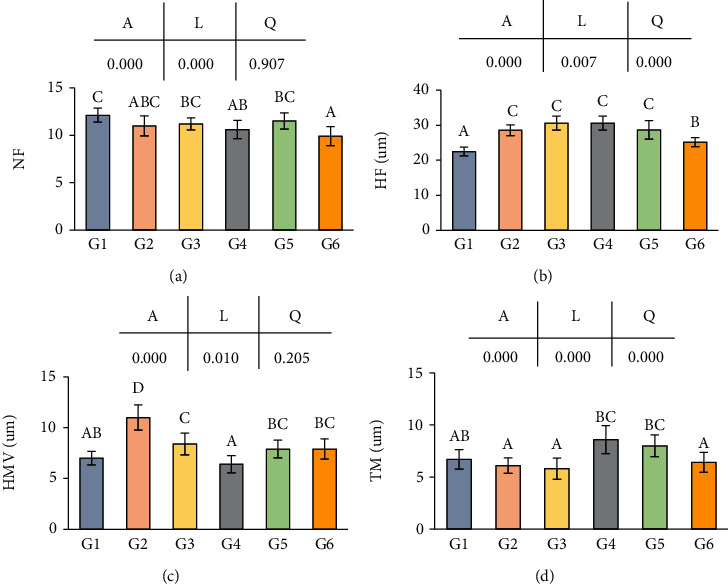
The intestine micromorphology of *Eriocheir sinensis* fed with different levels of niacin. (a) NF: number of folds; (b) HF: height of fold; (c) HMV: height of microvillus; (d) TM: thickness of muscularis. The values are the means ± S.D. (*n* = 3). Different letters indicate significant differences (*p* < 0.05). *A*: ANOVA; *L*: linear trend; *Q*: quadratic trend.

Effects of dietary niacin levels on histomorphology of the intestines in crabs are shown in Figure 4. There was an overall decreasing trend in NF of crab intestines as dietary niacin levels increased, the highest in G1 and the lowest in G6 (*p* < 0.05) (Figure 4(a)). The intestinal HF of crabs reached the highest in G3 and G4 which significantly increased with dietary niacin increasing from 0.89 to 587.59 mg/kg and significantly decreased with dietary niacin increasing from 784.85 to 1248.86 mg/kg (*p* < 0.05) (Figure 4(b)). Moreover, the intestinal HMV of crabs significantly increased initially and then significantly decreased when niacin content was less than 587.59 mg/kg (G4) (*p* < 0.05). However, when niacin content exceeded 587.59 mg/kg (G4), HMV significantly increased (*p* < 0.05), while no significant difference in HMV was found among G1, G5, and G6 (*p* > 0.05), as well as among G3, G5, and G6 (*p* > 0.05) (Figure 4(c)). The intestinal TM in G2 and G3 was significantly lower than that in G4 and G5 (*p* < 0.05), while no significant difference in TM was found among G1, G2, G3, and G6 (*p* > 0.05) (Figure 4(d)). Linearly, a trend was found between the dietary niacin levels and the dependent variables including the NF and HMV of crab intestines. Quadratic trend was found between dietary niacin levels and the dependent variables including HF and TM.

### 3.4. Antioxidant and Immunity Parameters of the Hepatopancreas

**Figure 5:**
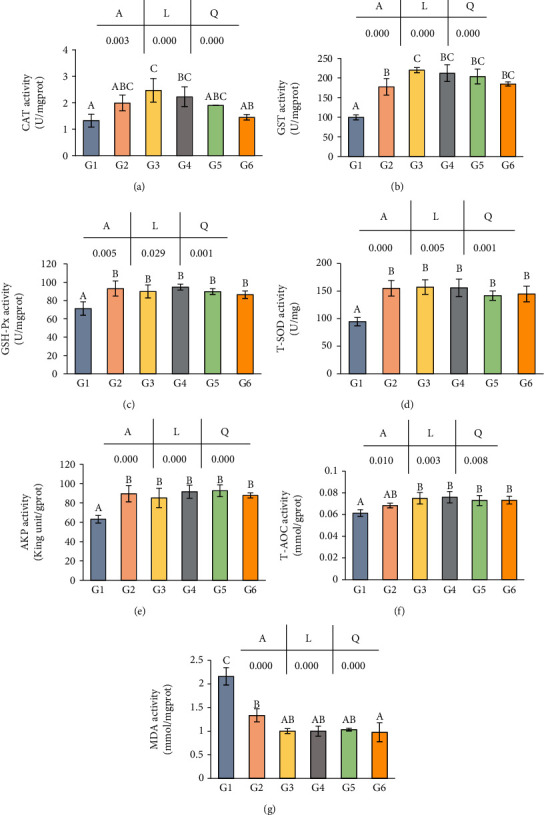
The activities of CAT, MDA, T-AOC, GST, GSH-Px, T-SOD, and AKP value in hepatopancreas of *Eriocheir sinensis* fed with different levels of niacin. (a) CAT: acid phosphatase; (b) GST: glutathione s-transferase; (c) GSH-Px: glutathione peroxidase; (d) T-SOD: total superoxide dismutase; (e) AKP: alkaline phosphatase; (f) T-AOC: total antioxidant capacity; (g) MAD: malondialdehyde. The values are the means ± S.D. (*n* = 3). Different letters indicate significant differences (*p* < 0.05). *A*: ANOVA; *L*: linear trend; *Q*: quadratic trend.

Effects of dietary niacin levels on the immune response in hepatopancreas of crabs are shown in Figure 5. CAT and GST activities were significantly increased with the niacin level increased from 0.89 to 347.05 mg/kg, while tending to decrease from 347.05 to 1248.86 mg/kg (*p* < 0.05) (Figures 5(a) and 5(b)). GSH-Px, T-SOD, and AKP activities in the control group were significantly lower than those in other five groups (*p* < 0.05), while no significant difference was found among niacin supplemented groups (*p* > 0.05) (Figures 5(c)–5(e)). T-AOC activity of G1 was significantly lower than that of G3, G4, G5, and G6 (*p* < 0.05), while no significant difference was found between G1 and G2, as well as groups fed with dietary niacin more than 170.54 mg/kg (*p* > 0.05) (Figure 5(f)). Additionally, MDA value in G1 was significantly higher than that in G2 (*p* < 0.05), and G2 was significantly higher than the other four groups, while no significant difference was found among G3, G4, G5, and G6 (*p* > 0.05) (Figure 5(g)). In addition, there were significantly linear and quadratic trends between the dietary niacin levels and the activities of CAT, GST, GSH-Px, T-SOD, AKP, T-AOC, and MDA.

## 4. Discussion

The present results indicated that niacin supplementation with appropriate amounts improved the growth performance; the maximum SGR could be achieved with dietary 419.4 mg/kg niacin. Meanwhile, according to the relationship between the hepatopancreas niacin content and dietary niacin level, the maximum niacin deposition in the hepatopancreas could be achieved with dietary 415.2 mg/kg niacin. Based on the results of crabs' growth performance and niacin deposition in hepatopancreas, the niacin requirements of juvenile *E. sinensis* were suggested to be 419.4 mg/kg and 415.2 mg/kg, respectively, which were significantly higher than those of grass shrimp [3], Indian white prawn [2], and Pacific white shrimp [4], but similar to for the requirements of kuruma prawn [11] and Chinese prawn [12]. Compared with fish, the niacin requirements of juvenile *E. sinensis* are much higher. This difference might be related to the following factors: firstly, *E. sinensis* is known to be slow feeders; it may take about 1 hour to accomplish feeding, especially under laboratory conditions; secondly, although niacin is physiochemically stable, it is prone to leach out of the pellets because of its water solubility. This has been confirmed by the leaching test, in which the niacin content in diets was reduced to 53.70-60.85% after being soaked in water for 1 h. In addition, niacin is involved in coenzyme systems of carbohydrate metabolism in energy-generating systems of intermediary metabolism where food (or feed) material is oxidized to furnish heat for physiological functions to maintain homoeostasis for body temperature in homeotherms [5]. Shiau and Suen showed that the niacin requirements of hybrid tilapia are much higher with dextrin (121 mg/kg) as the carbohydrate source than with monosaccharide glucose (26 mg/kg) as the carbohydrate source [28]. These results indicated that complex carbohydrates in the diet may increase the requirement for niacin. In the present study, we use dextrin as the carbohydrate source which may be one of the reasons for the high niacin requirement.

The growth performance and nutrient deposition in tissues are generally applied to determine the nutrient requirement of aquatic animals. In the present study, the niacin requirement of *E. sinensis* concluded by the SGR is higher than that by liver niacin deposition, which is similar to that reported in juvenile blunt snout bream [29]. Notably, only trace amounts of niacin were deposited in the hepatopancreas in the G1 and G2 groups. This might be due to the insufficient niacin supplementation given to meet the growth and physiological needs of *E. sinensis*. With the dietary niacin increased, the deposition of niacin in the hepatopancreas was significantly increased and reached 115.64 mg/kg.

Some fish have the ability to convert tryptophan to niacin and reduce the need for exogenous niacin [28]. Moreover, some gut bacteria have been reported to synthesize niacin which also contributes to reducing feed supplementation [30, 31]. In the present study, *E. sinensis* in the control group did not show visible deficiency symptoms, apart from the lower growth performance, which was in agreement with that reported for the giant tiger prawn [3] and the Pacific white shrimp [4]. However, the ability to self-supply niacin has not been reported in crabs, which needs further investigation.

Intestines play an important role in digestion and absorption of nutrients. Some indices related to mucosal folds are usually analyzed to evaluate the effects of nutrients on feed utilization. In the present study, increased levels of dietary niacin positively affected the intestinal morphology of juvenile *E. sinensis*, including NF, HF, TM, and HMV, which demonstrated that appropriate dietary niacin enhanced the surface area of the intestines. However, we did not find that appropriate levels of niacin significantly increased the feed utilization efficiency of crabs.

Because of lacking specific immune systems, *E. sinensis* is extremely dependent on the nonspecific immune system [32]. AKP plays significant roles in regulating intracellular processes involved in cell cycle, growth, apoptosis, and signal transduction pathways in organisms. In this study, dietary niacin significantly increased AKP activity in *E. sinensis*, which was consistent with that found in juvenile golden pompano [10]. Moreover, T-AOC is an index reflecting the overall capacity of the body's antioxidant enzymatic (e.g., SOD, CAT, and GSH-Px) and nonenzymatic systems (e.g., vitamins, carotenoids) [33]. The current study found that niacin was able to significantly increase the T-AOC of *E. sinensis*, which was consistent with the reports of juvenile hybrid sturgeon (*Acipenser schrenckii♂ × A. baeri♀*) [34] and golden pompano [10]. SOD and CAT are the main components of the enzymatic antioxidant system in aquatic animals. SOD is the protease that eliminates oxygen radicals in the body, mainly responsible for the conversion of O^2-^ to H_2_O_2_ and the removal of O^2-^ from the body, which is the key to the survival of aerobic organisms and defense against oxygen toxicity, so the SOD activity in tissues indirectly affects the ability of the body to scavenge oxygen radicals [4]. Moderate niacin intake increased T-SOD activity in juvenile *E. sinensis*, while excess niacin slightly reduced SOD activity, which is similar to the studies of hybrid sturgeon [33]. However, niacin did not affect the activity of SOD in white shrimp and blunt snout bream, and the reasons for this difference need further study. CAT catalyzes the decomposition of H_2_O_2_ into H_2_O and O^2-^ [4]. Excess niacin resulted in a decreased CAT activity of young grass carp [35]; the same result was found in the present study. GSH-Px is a widespread enzyme that catalyzes the breakdown of hydrogen peroxide in animals, producing water and O_2_ from the formed hydrogen peroxide and preventing further hydrolysis of peroxide to produce the harmful substance MDA [36]. In this study, the activities of GSH-Px were improved by increasing the level of dietary niacin in *E. sinensis*, and the MDA content was decreased, suggesting that niacin has a positive regulatory effect on the antioxidant capacity of juvenile *E. sinensis*.

## 5. Conclusion

Optimal dietary niacin can improve the growth performance, niacin deposition in hepatopancreas, antioxidant capacity, and nonspecific immunity of *E. sinensis*. Similarly, optimal dietary niacin positively changed the intestinal histomorphology by decreasing the NF and increasing the HF and TM of intestines in crabs. However, dietary niacin level did not affect the survival rate, feed conversion ratio, daily feeding rate, and molting frequency of crabs. Under the present experimental conditions, the optimum niacin requirement of juvenile *E. sinensis* was suggested to be 419.4 mg/kg, based on the SGR of crabs.

## Figures and Tables

**Table 1:** Ingredient formulation and proximate composition of the six experimental diets fed to *Eriocheir sinensis* (% dry matter).

Ingredients	Content (%)					
	G1	G2	G3	G4	G5	G6
Casein (vitamin-free)	45.00	45.00	45.00	45.00	45.00	45.00
Dextrin	23.00	23.00	23.00	23.00	23.00	23.00
Carboxymethyl cellulose	13.12	13.10	13.08	13.06	13.04	13.00
Choline chloride	0.50	0.50	0.50	0.50	0.50	0.50
Cholesterol	0.50	0.50	0.50	0.50	0.50	0.50
Lecithin	1.50	1.50	1.50	1.50	1.50	1.50
Dimethyl-beta-propiothetin	0.30	0.30	0.30	0.30	0.30	0.30
Amino acid premix^a^	0.38	0.38	0.38	0.38	0.38	0.38
Mineral premix^b^, niacin-free	3.00	3.00	3.00	3.00	3.00	3.00
Vitamin premix^c^	2.00	2.00	2.00	2.00	2.00	2.00
Fish oil	4.00	4.00	4.00	4.00	4.00	4.00
Soybean oil	2.00	2.00	2.00	2.00	2.00	2.00
Guar gum	1.50	1.50	1.50	1.50	1.50	1.50
Calcium dihydrogen phosphate	2.00	2.00	2.00	2.00	2.00	2.00
L-Ascorbic acid-2-trisodium phosphate	0.50	0.50	0.50	0.50	0.50	0.50
D-*α*-Tocopherol acetate	0.10	0.10	0.10	0.10	0.10	0.10
Inositol	0.60	0.60	0.60	0.60	0.60	0.60
Niacin	0.00	0.2	0.4	0.6	0.8	1.2
Total	100	100	100	100	100	100
Proximate composition (% dry matter)						
Moisture	8.78	8.87	8.66	8.67	8.40	8.62
Crude protein	42.38	44	44.19	42.53	42.53	42.78
Crude lipid	5.36	5.2	5.26	5.29	5.31	5.31
Ash content	6.83	6.77	6.45	6.7	6.69	6.51
Niacin (mg/kg diet)	0.89	170.54	347.05	587.59	784.85	1248.86

^a^Amino acid premix (per 1 kg diet): arginine, 1.8 g; valine, 0.9 g. ^b^Mineral premix (per 1 kg diet): Ca (H_2_PO_4_)_2_, 10 g; MgSO_4_·7H_2_O, 2.4 g; KCl, 4.5 g; NaCl, 2.1 g; FeSO_4_·H_2_O, 155 mg; CuSO_4_·5H_2_O, 40 mg; ZnSO_4_·H_2_O, 80 mg; Mn·SO_4_·H_2_O, 30 mg; KI, 11.7 mg; CoCl_2_·6H_2_O, 4.8 mg; Na_2_SeO_3_, 2.4 mg. ^c^Vitamin premix (per 1 kg diet): vitamin A, 10000 IU; vitamin D, 2500 IU; vitamin K, 64 mg; thiamine, 60 mg; riboflavin, 250 mg; pyridoxine, 60 mg; calcium pantothenate, 240 mg; folic acid, 12 mg; biotin, 50 mg; cyanocobalamin, 4 mg.

**Table 2:** Growth performance, survival rate, and feed utilization of *Eriocheir sinensis* fed with different levels of niacin.

	Diets						*Pr* > *F*^a^		
	G1	G2	G3	G4	G5	G6	*A*	*L*	*Q*
_*W*i_ (g)	1.14 ± 0.03	1.14 ± 0.01	1.14 ± 0.01	1.14 ± 0.02	1.14 ± 0.02	1.14 ± 0.03	1.000	1.000	1.000
*W*_t_ (g)	2.62 ± 0.12*a*	2.81 ± 0.09*ab*	3.10 ± 0.05*bc*	3.21 ± 0.17*c*	3.06 ± 0.06*bc*	3.05 ± 0.08*bc*	0.000	0.000	0.000
WGR (%)	130.18 ± 10.99*a*	146.55 ± 7.96*ab*	171.59 ± 4.05*bc*	181.59 ± 15.23*c*	168.23 ± 5.70*bc*	167.49 ± 7.07*bc*	0.000	0.000	0.000
SGR (%/day)	1.19 ± 0.07*a*	1.28 ± 0.05*ab*	1.42 ± 0.02*c*	1.47 ± 0.08*c*	1.41 ± 0.03*bc*	1.40 ± 0.04*bc*	0.000	0.000	0.000
SR (%)	85.14 ± 3.2	88.89 ± 5.56	88.89 ± 0.00	96.30 ± 6.41	92.59 ± 3.20	88.89 ± 5.56	0.131	0.117	0.052
FCR	2.41 ± 0.29	2.29 ± 0.12	2.40 ± 0.05	2.43 ± 0.17	2.52 ± 0.13	2.59 ± 0.13	0.358	0.064	0.316
DFR (%)	3.36 ± 0.16	3.31 ± 0.22	2.77 ± 0.15	2.80 ± 0.38	3.11 ± 0.11	3.08 ± 0.13	0.056	0.117	0.017

Abbreviations: *W*_i_: initial individual weight; *W*_t_: final individual weight; WGR: weight gain rate; SGR: specific growth rate; SR: survival rate; FCR: feed conversion ratio; DFR: daily feeding rate. Data in the same line with different superscript letters are significantly different (*p* < 0.05) as determined by Tukey's test. The values are the means ± S.D. (*n* = 3). ^a^Significance probability associated with the *F*-statistic. *A*: the variance analyzed by one-way ANOVA; *L*: linear trend analyzed by orthogonal polynomial contrasts; *Q*: quadratic trend analyzed by orthogonal polynomial contrasts.

## Data Availability

Data is available on request.
